# Minimal Access vs Conventional Nipple-Sparing Mastectomy

**DOI:** 10.1001/jamasurg.2024.2977

**Published:** 2024-08-14

**Authors:** Joo Heung Kim, Jai Min Ryu, Soong June Bae, Beom Seok Ko, Jung Eun Choi, Ku Sang Kim, Chihwan Cha, Young Jin Choi, Hye Yoon Lee, Sang Eun Nam, Zisun Kim, Young-Joon Kang, Moo Hyun Lee, Jong Eun Lee, Eunhwa Park, Hyuk Jai Shin, Min Kyoon Kim, Hee Jun Choi, Seong Uk Kwon, Nak-Hoon Son, Hyung Seok Park, Jeeyeon Lee

**Affiliations:** 1 Department of Surgery, Yongin Severance Hospital, Yonsei University College of Medicine, Yongin, Korea; 2 Department of Surgery, Samsung Medical Center, Sungkyunkwan University School of Medicine, Seoul, Korea; 3 Department of Surgery, Gangnam Severance Hospital, Yonsei University College of Medicine, Seoul, Korea; 4 Department of Surgery, Asan Medical Center, University of Ulsan College of Medicine, Seoul, Korea; 5 Department of Surgery, Yeungnam University College of Medicine, Daegu, Korea; 6 Department of Surgery, Kosin University College of Medicine, Gospel Hospital, Busan, Korea; 7 Department of Surgery, Hanyang University Seoul Hospital, Hanyang University College of Medicine, Seoul, Korea; 8 Department of Surgery, Chungbuk National University Hospital, Cheongju, Korea; 9 Department of Surgery, Korea University Ansan Hospital, Ansan, Korea; 10 Department of Surgery, Konkuk University School of Medicine, Seoul, Korea; 11 Department of Surgery, Soonchunhyang University Bucheon Hospital, Bucheon, Korea; 12 Department of Surgery, Incheon St. Mary’s Hospital, The Catholic University of Korea, Incheon, Korea; 13 Department of Surgery, Keimyung University School of Medicine, Daegu, Korea; 14 Department of Surgery, Soonchunhyang University Cheonan Hospital, Cheonan, Korea; 15 Department of Surgery, Dong-A University Hospital, Dong-A University College of Medicine, Busan, Korea; 16 Department of Surgery, Myongji Hospital, Hanyang University Medical Center, Goyang, Korea; 17 Department of Surgery, Chung-Ang University Hospital, Seoul, Korea; 18 Department of Surgery, Samsung Changwon Hospital, Sungkyunkwan University School of Medicine, Changwon, Korea; 19 Department of Surgery, Konyang University Hospital, Daejeon, Korea; 20 Department of Statistics, Keimyung University, Daegu, Korea; 21 Department of Surgery, Yonsei Cancer Center, Yonsei University College of Medicine, Seoul, Korea; 22 Department of Surgery, School of Medicine, Kyungpook National University, Kyungpook National University Chilgok Hospital, Daegu, Republic of Korea

## Abstract

**Importance:**

While nipple-sparing mastectomy (NSM) for breast cancer was only performed using the open method in the past, its frequency using endoscopic and robotic surgical instruments has been increasing rapidly. However, there are limited studies regarding postoperative complications and the benefits and drawbacks of minimal access NSM (M-NSM) compared with conventional NSM (C-NSM).

**Objective:**

To examine the differences in postoperative complications between C-NSM and M-NSM.

**Design, Setting, Participants:**

This was a retrospective multicenter cohort study enrolling 1583 female patients aged 19 years and older with breast cancer who underwent NSM at 21 university hospitals in Korea between January 2018 and December 2020. Those with mastectomy without preserving the nipple-areolar complex (NAC), clinical or pathological malignancy in the NAC, inflammatory breast cancer, breast cancer infiltrating the chest wall or skin, metastatic breast cancer, or insufficient medical records were excluded. Data were analyzed from November 2021 to March 2024.

**Exposures:**

M-NSM or C-NSM.

**Main Outcomes and Measures:**

Clinicopathological factors and postoperative complications within 3 months of surgery were assessed. Statistical analyses, including logistic regression, were used to identify the factors associated with complications.

**Results:**

There were 1356 individuals (mean [SD] age, 45.47 [8.56] years) undergoing C-NSM and 227 (mean [SD] age, 45.41 [7.99] years) undergoing M-NSM (35 endoscopy assisted and 192 robot assisted). There was no significant difference between the 2 groups regarding short- and long-term postoperative complications (<30 days: C-NSM, 465 of 1356 [34.29%] vs M-NSM, 73 of 227 [32.16%]; *P* = .53; <90 days: C-NSM, 525 of 1356 [38.72%] vs M-NSM, 73 of 227 [32.16%]; *P* = .06). Nipple-areolar complex necrosis was more common in the long term after C-NSM than M-NSM (C-NSM, 91 of 1356 [6.71%] vs M-NSM, 5 of 227 [2.20%]; *P* = .04). Wound infection occurred more frequently after M-NSM (C-NSM, 58 of 1356 [4.28%] vs M-NSM, 18 of 227 [7.93%]; *P* = .03). Postoperative seroma occurred more frequently after C-NSM (C-NSM, 193 of 1356 [14.23%] vs M-NSM, 21 of 227 [9.25%]; *P* = .04). Mild or severe breast ptosis was a significant risk factor for nipple or areolar necrosis (odds ratio [OR], 4.75; 95% CI, 1.66-13.60; *P* = .004 and OR, 8.78; 95% CI, 1.88-41.02; *P* = .006, respectively). Conversely, use of a midaxillary, anterior axillary, or axillary incision was associated with a lower risk of necrosis (OR for other incisions, 32.72; 95% CI, 2.11-508.36; *P* = .01). Necrosis occurred significantly less often in direct-to-implant breast reconstruction compared to other breast reconstructions (OR, 2.85; 95% CI, 1.11-7.34; *P* = .03).

**Conclusions and Relevance:**

The similar complication rates between C-NSM and M-NSM demonstrates that both methods were equally safe, allowing the choice to be guided by patient preferences and specific needs.

## Key Points

**Question:**

Does the incidence of postoperative complications differ between conventional nipple-sparing mastectomy (C-NSM) and minimal access nipple-sparing mastectomy (M-NSM)?

**Findings:**

In this case-control study of 1356 individuals who underwent C-NSM and 227 who underwent M-NSM. There was no significant difference between the 2 groups regarding short- and long-term postoperative complications.

**Meaning:**

The incidence of complications following M-NSM was comparable to that following C-NSM, indicating its potential as a viable option for breast cancer treatment.

## Teaser

This case-control study evaluates outcomes following minimal access nipple-sparing mastectomy vs conventional nipple-sparing mastectomy.

## Introduction

Breast cancer is the most common type of cancer among women worldwide.^1^ The widespread uptake of breast cancer screening in many countries has led to a considerable increase in patients undergoing breast conserving surgery for early breast cancer. Even among patients diagnosed with advanced breast cancer, a substantial proportion of patients undergo breast conserving surgery after receiving neoadjuvant treatment; however, the rate of total mastectomy has remained greater than 30%.^2^ Furthermore, with an increased understanding of the *BRCA1/2* variant, the frequency of prophylactic mastectomy has also increased.^3^

Nipple-sparing mastectomy (NSM) is increasingly performed owing to its superior esthetic outcomes compared with those of conventional mastectomy.^4^ However, conventional NSM (C-NSM) leaves a large visible scar on the breast and there is a high potential risk of necrosis of the nipple-areolar complex (NAC), depending on the approach used.^5^ Although the inframammary fold (IMF) approach eliminates visible scarring, it has the disadvantage of providing insufficient visual access; in addition, approaching the superior pole of the breast and the axillary area is challenging with this approach.^6^

Minimal access NSM (M-NSM) such as endoscopy-assisted or robot-assisted NSM refers to a surgical procedure for NSM that uses endoscopic or robotic devices.^7,8^ M-NSM allows the creation of relatively short incisions in less visible areas. M-NSM can be performed by inflating the breast with CO_2_ to create space or by using a gasless technique that retracts the skin via an incision.^9^ This method helps to overcome the disadvantages of C-NSM.^10^ The early experience of robotic surgery in Korea reported by the Korea Robot-Endoscopy Minimal Access Breast Surgery Study Group (KoREa-BSG) demonstrated the potential usefulness of M-NSM in patients with breast cancer,^11^ and several studies of robot-assisted NSM have been reported.^12,13,14,15^ However, some surgical oncologists have expressed apprehension regarding their inability to palpate breast tissue or lesions during M-NSM, especially in robot-assisted NSM.^16^ This limitation may increase the risk of complications, such as skin or NAC necrosis, which are critical for aesthetic outcomes. Moreover, extensive research on the advantages and disadvantages of M-NSM is lacking. This study aimed to compare postoperative complications between C-NSM and M-NSM and to identify factors that may influence such differences.

## Methods

### Patient Selection

This retrospective multicenter study included 1583 breast cancer patients who underwent C-NSM or M-NSM between January 2018 and December 2020 across 21 institutions in the Republic of Korea. This study was approved by the institutional review board of the Yongin Severance Hospital of Yonsei University. Due to the retrospective analysis, the requirement for patient consent was waived by the IRB.

The inclusion criterion for this study was female patients aged 19 years and older who underwent NSM for breast cancer irrespective of the location of the skin incision. The exclusion criteria were mastectomy without preserving the NAC, clinical or pathological malignancy in the NAC, inflammatory breast cancer, breast cancer infiltrating the chest wall or skin, metastatic breast cancer, and insufficient medical records. The patients were classified into 2 groups based on the surgical method: C-NSM (n = 1356) and M-NSM (n = 227). M-NSM was performed as endoscopy-assisted NSM in 35 individuals and robot-assisted NSM in 192 individuals. The analysis of complications was limited to complications that occurred within 3 months after surgery.

### Clinicopathologic Variables

Clinicopathological variables, including age, body mass index, menopausal status, breast size, breast ptosis, history of smoking, medical history, germline variant status, adjuvant treatment, TNM stage, histological grade, histological type, estrogen receptor, progesterone receptor, human epidermal growth factor (HER) 2, and *Ki-67*, were collected. Surgical variables, including specimen weight, type of breast reconstruction, location of surgical incision, incision length, amount of intraoperative bleeding, and operation time, were analyzed. TNM staging was performed according to the anatomic stage of the American Joint Committee on Cancer, 8th edition.^17^ Estrogen receptor and progesterone receptor positivity were defined as 1% or greater nuclear staining in immunohistochemistry. *HER2* positivity was defined as either 3+ staining in immunohistochemistry or 2+ staining in immunohistochemistry with confirmed amplification in fluorescence in situ hybridization or silver in situ hybridization, according to the guidelines of the American Society of Clinical Oncology and College of American Pathologists.^18^

C-NSM was performed through various skin incisions, including the upper outer radial, IMF, periareolar and extension, elliptical, periareolar only, horizontal, midaxillary or anterior axillary, inferior radial, and axillary incision. The M-NSM incision was made in the lateral chest, anterior axillary line, or midaxillary line. M-NSM involved the use of endoscopic devices, such as advanced energy devices, endoscopic forceps, endoscopic scissors, fiberoptic retractors, and self-retractors. For the gas-inflated technique, multiple single-access ports, such as Glove port (Nelis), Octo-port (Dalim SurgNet Corp), Uni-port (Dalim), Gelpoint Mini (Applied Medical), Oneport (Tebah), Lapsingle (Sejong Medical), and hand-made glove port were used for maintaining gas insufflation. For the gasless technique, Chung self-retractors or fiberoptic retractors were used to create and maintain working space. Previous researchers have described detailed M-NSM techniques.^7,9,19,20,21^

### Postoperative Complications

Postoperative complications were categorized as short term (<30 days) and long term (<90 days). Postoperative complications were also classified according to the Clavien-Dindo classification.^22^ Complications of grade IIIb or higher based on the Clavien-Dindo classification include those requiring intervention under general anesthesia, life-threatening complications, and those leading to death. The records regarding postoperative complications, including NAC necrosis, skin necrosis, breast infection, wound dehiscence, bleeding or hematoma, postoperative seroma, and loss of breast implants, were collected. If more than 2 complications occurred in 1 patient, all complications were recorded and included in the analysis. NAC necrosis after surgery was classified into 6 stages (A-F) based on the extent and severity of necrosis within the nipple and areola^23^ (eFigure 1 in Supplement 1). The severity of skin necrosis was assessed by combining depth scores A-D and area scores 0-3.^24^ Depth of skin necrosis was defined as follows: A, no evidence of necrosis; B, only color change; C, partial-thickness necrosis; and D, full-thickness necrosis. The area of skin necrosis was scored according to the percentage of involved skin (score 0, <1%; score 1, 1%-10%; score 2, 11%-30%; and score 3, >30%) (eFigure 2 in Supplement 1).

### Statistical Analysis

The Shapiro-Wilk test was used to assess the normality of distribution of continuous variables. Continuous variables were expressed as means with SDs and between-group differences were assessed for statistical significance using the *t* test. Categorical variables were expressed as frequencies with percentages and between-group differences were assessed using the χ^2^ or Fisher exact test.

For postoperative complications in the short term, statistical analysis could not be properly performed due to the low frequency of occurrence. However, for postoperative complications in the long term, statistical analysis was conducted differently for each factor. Logistic regression was used to determine the factors affecting NAC necrosis. Firth regression can overcome the problem of the lack of a finite confidence interval, which often occurs in regressions with a low number of events. Therefore, Firth regression was considered due to the low number of necrotic events. Univariate logistic regression analysis was performed to identify factors associated with necrosis. Subsequently, multivariate logistic regression was conducted to adjust for covariates in the model. The results are presented as odds ratios (ORs) and 95% CIs. Statistical analyses were performed using SAS version 9.4 (SAS Institute). Two-tailed *P *values <.05 were considered indicative of statistical significance.

## Results

Among the 1583 patients included in the study, 1356 (mean [SD] age, 45.47 [8.56] years) were in the C-NSM group and 227 (mean [SD] age, 45.41 [7.99] years) in the M-NSM group (35 endoscopy assisted and 192 robot assisted). There were no significant differences between the 2 groups in terms of clinicopathological variables, except for menopausal status, grade of breast ptosis, *BRCA1/2* variant, and neoadjuvant chemotherapy. The M-NSM group had a significantly higher proportion of premenopausal patients (C-NSM, 904 of 1356 [66.67%]; M-NSM, 167 of 227 [73.57%]; *P* = .02), as well as a higher proportion of nonptotic breasts (C-NSM, 424 of 1356 [31.27%]; M-NSM, 150 of 227 [66.08%]; *P* < .001). There were no significant differences between groups for T stage, N stage, estrogen receptor, progesterone receptor, HER2 gene status, or histologic grade (Table 1). The M-NSM group had significantly more cases of bilateral surgery (C-NSM, 128 of 1356 [9.44%]; M-NSM, 47 of 227 [20.70%]; *P* < .001), a higher rate of performing sentinel lymph node biopsy (C-NSM, 1267 of 1356 [93.44%]; M-NSM, 221 of 227 [97.36%]; *P* = .01), larger implant volume (mean [SD], C-NSM, 287.99 [189.36] mL^3^; M-16 NSM, 339.95 [114.75] mL^3^; *P* < .001), and smaller final incision length (mean [SD], C-NSM, 76.15 [17.55] mm; M-NSM, 48.61 [11.89] mm; *P* < .001). Regarding the patient’s position during surgery, while the 90° arm extension was most frequently used in the C-NSM group (n = 894 [65.93%]), the raising arm position was most frequently used in the M-NSM group (n = 113 [49.78%]) (*P* < .001). The most frequent incision method in C-NSM was upper outer radial incision (n = 468, 34.51%), whereas that in the M-NSM was midaxillary or anterior axillary incision (n = 177 [77.97%]) (*P* < .001). In the subcutaneous flap dissection for NSM, while electrocauterization alone was most preferred in C-NSM (n = 697 [51.40%]), a combination of hydrodissection and electrocauterization was most frequently used in M-NSM (n = 187 [82.38%]) (*P* < .001). The operative time was significantly longer in the M-NSM group than in the C-NSM group (mean [SD], C-NSM, 116.01 [47.21] minutes; M-NSM, 146.94 [47.09] minutes; *P* < .001); however, there was no significant between-group difference in specimen weight. There was no significant difference between 2 groups in the amount of intraoperative blood loss, but the volume of serous fluid drained after surgery was significantly greater in the M-NSM group (mean [SD], C-NSM, 959.33 [657.59] mL^3^; M-NSM, 1333.28 [859.29] mL^3^; *P* < .001), and the duration of placement of drainage tube was significantly longer in M-NSM than C-NSM (mean [SD], C-NSM, 12.61 [4.11] days; M-NSM, 16.88 [9.84] days; *P* < .001) (Table 2).

**Table 1.** Clinicopathologic Characteristics of Patients With Breast Cancer Who Underwent Conventional Nipple-Sparing Mastectomy (C-NSM) or Minimal Access Nipple-Sparing Mastectomy (M-NSM)

Characteristic	No. (%)		*P* value
	C-NSM (n = 1356)	M-NSM (n = 227)	
Age, mean (SD), y	45.47 (8.56)	45.41 (7.99)	.92
BMI, mean (SD)	22.71 (3.15)	22.52 (3.04)	.39
Menopausal status			
Premenopausal	904 (66.67)	167 (73.57)	.02
Postmenopausal	291 (21.46)	34 (14.98)	
Unknown	161 (11.87)	26 (11.45)	
Ptosis			
Normal	424 (31.27)	150 (66.08)	<.001
Mild	172 (12.68)	31 (13.66)	
Moderate	145 (10.69)	5 (2.2)	
Severe	79 (5.83)	2 (0.88)	
Pseudoptosis	14 (1.03)	2 (0.88)	
Unknown	522 (38.5)	37 (16.3)	
Smoking history			
Nonsmoking	866 (63.86)	208 (91.63)	.24
Smoking	40 (2.95)	14 (6.17)	
Unknown	430 (31.71)	5 (24.23)	
*BRCA* variant			
No test	1080 (79.65)	143 (63)	<.001
Negative	174 (12.83)	67 (29.52)	
Positive	85 (6.27)	11 (4.85)	
Variant of unknown significance	26 (1.92)	6 (2.64)	
pT stage			
0 or In situ	257 (18.95)	63 (27.75)	.15
1	516 (38.05)	117 (51.54)	
2	240 (17.7)	36 (15.86)	
3	26 (1.92)	6 (2.64)	
Unknown	317 (23.38)	5 (2.2)	
pN stage			
0 or Micrometastasis	825 (60.84)	193 (85.02)	.06
1	169 (12.46)	23 (10.13)	
2	30 (2.21)	3 (1.32)	
3	9 (0.66)	3 (1.32)	
Unknown	323 (23.82)	5 (2.2)	
Histologic grade			
Well	175 (12.91)	33 (14.54)	.31
Moderate	680 (50.15)	110 (48.46)	
Poor	267 (19.69)	34 (14.98)	
Unknown	234 (17.26)	50 (22.03)	
Estrogen receptor			
Negative	291 (21.46)	41 (18.06)	.28
Positive	1058 (78.02)	182 (80.18)	
Unknown	7 (0.52)	4 (1.76)	
Progesterone receptor			
Negative	398 (29.35)	56 (24.67)	.18
Positive	950 (70.06)	167 (73.57)	
Unknown	8 (0.59)	4 (1.76)	
*HER2* gene			
Negative	959 (70.72)	142 (62.56)	.12
Equivocal	144 (10.62)	29 (12.78)	
Positive	245 (18.07)	50 (22.03)	
Unknown	8 (0.59)	6 (2.64)	
SISH or FISH			
Negative	230 (16.96)	62 (27.31)	.16
Positive	47 (3.47)	7 (3.08)	
Not applicable	1079 (79.52)	158 (69.6)	
Ki-67 index, mean (SD), %	20.01 (19.71)	19.7 (16.94)	.81
Neoadjuvant chemotherapy			
No	1135 (83.7)	204 (89.87)	.009
Yes	218 (16.08)	21 (9.25)	
Unknown	3 (0.22)	2 (0.88)	
Adjuvant chemotherapy			
No	921 (67.92)	159 (70.04)	.47
Yes	428 (31.56)	66 (29.07)	
Unknown	7 (0.52)	2 (0.88)	
Adjuvant radiotherapy			
No	1096 (80.83)	189 (83.26)	.33
Yes	252 (18.58)	36 (15.86)	
Unknown	8 (0.59)	2 (0.88)	

Abbreviations: BMI, body mass index (calculated as weight in kilograms divided by height in meters squared); *HER2*, human epidermal growth factor; FISH, fluorescence in situ hybridization; pN, pathological node; pT, pathological stage; SISH, silver in situ hybridization; VUS, variant of unknown significance.

**Table 2.** Comparative Analysis of Surgical Procedures and Outcomes in the Conventional Nipple-Sparing Mastectomy (C-NSM) and Minimal Access Nipple-Sparing Mastectomy (M-NSM) Groups

Variable	No. (%)		*P *value
	C-NSM (n = 1356)	M-NSM (n = 227)	
Risk-reducing surgery			
No	1348 (99.41)	226 (99.56)	>.99
Yes	8 (0.59)	1 (0.44)	
Surgical extent			
Unilateral	1228 (90.56)	180 (79.3)	<.001
Bilateral	128 (9.44)	47 (20.7)	
Arm position			
Raising arm	17 (1.25)	113 (49.78)	<.001
Laying down	129 (9.51)	34 (14.98)	
90° Extension	894 (65.93)	76 (33.48)	
Unknown	316 (23.3)	4 (1.76)	
Sentinel lymph node biopsy			
No	89 (6.56)	6 (2.64)	.01
Yes	1267 (93.44)	221 (97.36)	
Axillary lymph node dissection			
No	1135 (83.7)	199 (87.67)	.07
Yes	221 (16.3)	28 (12.33)	
Gas or gasless technique			
Gasless	NA	78 (34.36)	
Gas	NA	149 (65.64)	
Incision location			
Upper outer radial	468 (34.51)	6 (2.64)	<.001
Inframammary	332 (24.48)	10 (4.41)	
Periareolar and extension	153 (11.28)	1 (0.44)	
Elliptical	131 (9.66)	0	
Periareolar only	16 (1.18)	0	
Horizontal	28 (2.06)	0	
Mid or anterior axillary	5 (0.37)	177 (77.97)	
Inferior radial	2 (0.15)	0	
Axillary	0	12 (5.29)	
Other	210 (15.49)	18 (11.45)	
Initial incision size, mean (SD), cm	NA	45.33 (11.49)	<.001
Final incision size, mean (SD), cm	NA	48.61 (11.89)	<.001
Subcutaneous flap dissecting method			
Hydrodissection	21 (1.55)	7 (3.08)	<.001
Electrocauterization	697 (51.4)	33 (14.54)	
Both	212 (15.63)	187 (82.38)	
Unknown	426 (31.42)	NA	
Time for mastectomy, mean (SD), min	116.01 (47.21)	146.94 (47.09)	<.001
Specimen weight, mean (SD), g	361.71 (197.53)	347.88 (156.77)	.26
Reconstruction method			
Tissue expander	338 (24.93)	38 (16.74)	<.001
Direct to implant	682 (50.29)	167 (73.57)	
TRAM/DIEP flap	183 (13.5)	14 (6.17)	
LD flap	110 (8.11)	4 (1.76)	
Other	43 (3.17)	4 (1.76)	
Using energy device			
No	441 (32.52)	13 (5.73)	<.001
Yes	915 (67.48)	214 (95.96)	
Using acellular dermal matrix			
No	382 (28.17)	12 (5.29)	<.001
Yes	959 (70.72)	215 (94.71)	
Unknown	15 (11.06)	NA	
Implant volume, mean (SD), mL^3^	287.99 (189.36)	339.95 (114.75)	<.001
Amount of intraoperative bleeding, mean (SD), mL	170.55 (195.72)	136.24 (178.17)	.10
Total amount of drainage, mean (SD), mL	959.33 (657.59)	1333.28 (859.29)	<.001
Duration of drainage tube placement, mean (SD), d	12.61 (4.11)	16.88 (9.84)	<.001

Abbreviations: DIEP, deep inferior epigastric artery perforator; LD, latissimus dorsi myocutaneous; NA, not applicable; TRAM, transverse rectus abdominis musculocutaneous flap.

Based on Clavien-Dindo classification, 72 individuals (5.31%) in C-NSM and 7 (3.08%) in M-NSM developed grade IIIb or higher postoperative complications (*P* = .16). Although both short-term (<30 days) and long-term (<90 days) postoperative complications occurred more frequently in the C-NSM group, there was no statistical difference between the 2 groups (short term: C-NSM, 465 of 1356 [34.29%] vs M-NSM, 73 of 227 [32.16%]; *P* = .53; long term: C-NSM, 525 of 1356 [38.72%] vs M-NSM, 73 of 227 [32.16%], *P* = .06). Necrosis of the NAC occurred more frequently in the short term in the M-NSM group (C-NSM, 53 of 1356 [3.91%]; M-NSM, 20 of 227 [8.81%]); however, necrosis of the NAC in the long term occurred significantly more frequently in the C-NSM group (C-NSM, 91 of 1356 [6.71%]; M-NSM, 5 of 227 [2.20%]; *P* = .04). Wound infection occurred more frequently in the M-NSM group (short term: C-NSM, 22 of 1356 [1.62%] vs M-NSM, 17 of 227 [7.49%]; long term: C-NSM, 58 of 1356 [4.28%] vs M-NSM, 18 of 227 [7.93%]; *P* = .03). Postoperative seroma was significantly more frequently identified after C-NSM than M-NSM (C-NSM, 193 of 1356 [14.23%]; M-NSM, 21 of 227 [9.25%]; *P* = .04. In the C-NSM group, the amount of seroma was 3.68 times longer (*P* = .001), and the drainage period was 2.41 times greater (*P* < .001) than in the M-NSM group (Figure, Table 3).

**Figure.**
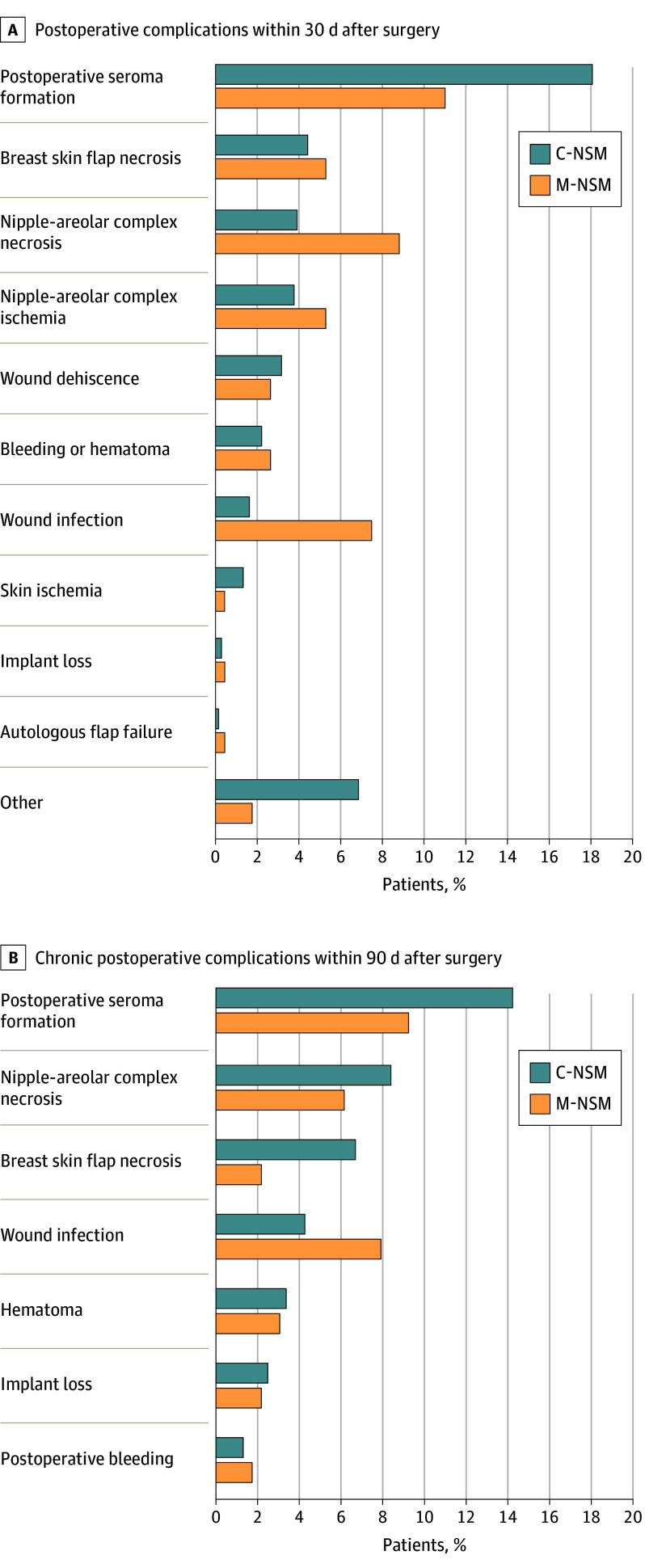
Comparison of Postoperative Complications After Conventional Nipple-Sparing Mastectomy (C-NSM) or Minimal Access Mastectomy (M-NSM)

**Table 3.** Postoperative Complications and Outcomes in the Conventional Nipple-Sparing Mastectomy (C-NSM) and Minimal Access Nipple-Sparing Mastectomy (M-NSM) Groups

Variable	No. (%)		*P* value
	C-NSM (n = 1356)	M-NSM (n = 227)	
Clavien-Dindo classification			
0-IIIa	1241 (91.52)	211 (92.95)	.16
IIIb-V	72 (5.31)	7 (3.08)	
Unknown	43 (3.17)	9 (3.96)	
Less than postoperative 30 d			
Occurrence of complications			
No	891 (65.71)	154 (67.84)	.53
Yes	465 (34.29)	73 (32.16)	
Postoperative seroma formation	245 (18.07)	25 (11.01)	NA
Breast skin flap necrosis	60 (4.42)	12 (5.29)	NA
Nipple-areolar complex necrosis	53 (3.91)	20 (8.81)	NA
Nipple-areolar complex ischemia	51 (3.76)	12 (5.29)	NA
Wound dehiscence	43 (3.17)	6 (2.64)	NA
Bleeding/hematoma	30 (2.21)	6 (2.64)	NA
Wound infection	22 (1.62)	17 (7.49)	NA
Skin ischemia	18 (1.33)	1 (0.44)	NA
Implant loss	4 (0.29)	1 (0.44)	NA
Autologous flap failure	2 (0.15)	1 (0.44)	NA
Other	93 (6.86)	4 (1.76)	NA
Less than postoperative 90 d			
Occurrence of complications			
No	831 (61.28)	154 (67.84)	.06
Yes	525 (38.72)	73 (32.16)	
Breast skin flap necrosis^a^			
A	1236 (91.15)	211 (92.95)	.62
B0	13 (0.96)	0	
B1	34 (2.51)	3 (1.32)	
B2	5 (0.37)	1 (0.44)	
B3	0	0	
C0	7 (0.52)	0	
C1	35 (2.58)	9 (3.96)	
C2	8 (0.59)	1 (0.44)	
C3	0	0	
D0	5 (0.37)	0	
D1	3 (0.22)	0	
D2	2 (0.15)	0	
D3	2 (0.15)	0	
Unknown	6 (0.44)	2 (0.88)	
Nipple-areolar complex necrosis^a^			
A	1203 (88.72)	200 (88.11)	.04
B	48 (3.54)	0	
C	20 (1.47)	2 (0.88)	
D	8 (0.59)	0	
E	5 (0.37)	0	
F	10 (0.74)	3 (1.32)	
Unknown	62 (4.57)	22 (9.69)	
Wound infection			
No	1293 (95.35)	208 (91.63)	.03
Minor infection	35 (2.58)	13 (5.73)	
Severe infection	23 (1.7)	5 (2.2)	
Unknown	5 (0.37)	1 (0.44)	
Postoperative bleeding			
No	1338 (98.67)	223 (98.24)	.61
Yes	18 (1.33)	4 (1.76)	
Hematoma			
No	1310 (96.61)	220 (96.92)	.81
Yes	46 (3.39)	7 (3.08)	
Postoperative seroma			
No	1163 (85.77)	206 (90.75)	.04
Yes	193 (14.23)	21 (9.25)	
Duration of seroma formation, mean (SD), d	51.33 (108.51)	13.93 (26.61)	.001
Total volume of aspirated seroma, mean (SD), mL	146.97 (270.1)	60.8 (53)	<.001
Implant loss			
No	1322 (97.49)	222 (97.8)	.78
Yes	34 (2.51)	5 (2.2)	

Abbreviation: NA, not applicable.

^a^ Definitions are shown in the eFigures in Supplement 1.

Presence of breast ptosis, whether mild or severe, was associated with a significantly higher risk of nipple or areolar necrosis (mild ptosis: OR, 4.75; 95% CI, 1.66-13.60; *P* = .004; severe ptosis: OR, 8.78; 95% CI, 1.88-41.02; *P* = .006). Direct-to-implant breast reconstruction (OR, 2.85; 95% CI, 1.11-7.34; *P* = .03) and midaxillary, anterior axillary, or axillary incisions (OR, 32.72; 95% CI, 2.11-508.36; *P* = .01) were associated with a significantly lower incidence of NAC necrosis compared to alternative reconstruction methods (Table 4).

**Table 4.** Risk Factors Associated With Nipple or Areolar Necrosis After Nipple-Sparing Mastectomy

Variable	Univariate logistic regression		Multivariate logistic regression	
	OR (95% CI)	*P* value	OR (95% CI)	*P* value
Breast ptosis				
Normal	1 [Reference]	NA	1 [Reference]	NA
Mild	3.65 (1.816-7.336)	.26	4.75 (1.66-13.60)	.003
Moderate	4.232 (2.034-8.805)	.14	2.11 (0.60-7.48)	.25
Severe	5.252 (2.235-12.344)	.06	8.78 (1.88-41.02)	.01
Pseudoptosis	1.013 (0.053-19.246)	.47	5.59 (0.16-197.35)	.34
History of smoking				
Never	1 [Reference]	NA	1 [Reference]	NA
Past	4.416 (1.494-13.055)	.07	6.63 (0.73-60.61)	.09
Current	1.876 (0.485-7.252)	.88	0.71 (0.04-12.59)	.81
Subcutaneous flap dissecting method				
Hydrodissection	1 [Reference]	NA	1 [Reference]	NA
Electrocauterization	1.565 (0.403-6.076)	.32	2.88 (0.58-14.39)	.20
Both	0.616 (0.323-1.176)	.12	0.94 (0.36-2.47)	.91
Reconstruction method				
Direct to implant	1 [Reference]	NA	1 [Reference]	NA
Other	1.79 (1.177-2.721)	.006	2.85 (1.11-7.34)	.03
Location of surgical incision				
Mid or anterior axillary or axillary	1 [Reference]	NA	1 [Reference]	NA
Other	5.096 (1.431-18.147)	.01	32.72 (2.11-508.36)	.01
Use of acellular dermal matrix				
No	1 [Reference]	NA	1 [Reference]	NA
Yes	0.502 (0.327-0.77)	.002	1.51 (0.51-4.49)	.46
Neoadjuvant chemotherapy				
No	1 [Reference]	NA	1 [Reference]	NA
Yes	1.025 (0.584-1.799)	.93	1.44 (0.47-4.46)	.52

Abbreviation: NA, not applicable.

No statistical difference in skin or NAC necrosis was observed between individuals who received C-NSM via IMF incision or M-NSM. However, the frequency of breast infection was significantly higher in the M-NSM group (C-NSM, 2 of 332 [0.60%]; M-NSM, 18 of 227 [7.93%]; *P* < .001), while the mean (SD) final length of incision was significantly longer in the C-NSM group using IMF incision than M-NSM (C-NSM, 83.62 [13.16] mm; M-NSM, 48.61 [11.89] mm; *P* < .001) (eTable in Supplement 1).

## Discussion

In this case-control study, we compared the surgical outcomes of M-NSM and C-NSM in patients with breast cancer. Although there were no significant differences in overall frequency of postoperative complications, M-NSM showed advantages, such as significantly lower incidence of NAC necrosis and seroma. Also, the length of incision was significantly shorter in M-NSM group. Interestingly, despite the more frequent use of hydrodissection, advanced energy devices, and acellular dermal matrix, the surgical time was approximately 30 minutes longer in M-NSM, whereas breast reconstruction surgery was longer by approximately 44 minutes in C-NSM. More than 73% of reconstructions after M-NSM used direct implantation methods compared with flap surgery for C-NSM, contributing to the difference in duration. Total surgical time was similar between groups.

The rate of NAC necrosis after C-NSM varies from 0% to 48%.^25,26,27,28,29,30,31^ In a study of 12 358 C-NSM procedures, the NAC necrosis rate was reported as 5.9%.^4^ In a multicenter study of robot-assisted NSM and C-NSM, NAC necrosis rates were 2.1% and 7.8%, respectively.^32^ Similarly, in this study, NAC necrosis rates in the long term were 2.20% and 6.71% in the M-NSM and C-NSM groups, respectively. However, the NAC necrosis rate in the short term in the M-NSM group was 8.81% higher than that of C-NSM. Robot-assisted NSM in South Korea was first reported in 2018,^20^ and many institutions have started this procedure for NSM since then.^11^ Therefore, this study included early experiences of M-NSM and the grades of NAC necrosis may not be clearly reported. However, given that there was no difference in NAC necrosis rate in the long term, it is likely that most were low grade, manageable, and improved quickly.

Multivariate analysis in this study adjusted for factors like breast ptosis, smoking history, flap dissection method, reconstruction method, incision location, acellular dermal matrix use, and neoadjuvant chemotherapy to identify influences on NAC necrosis. Independent factors found were incision location, reconstruction method, and ptosis. Using an axillary incision in NSM helps preserve blood supply to the NAC and skin, potentially reducing necrosis rates. This preservation of the blood supply may have contributed to the reduced incidence of NAC necrosis. In addition, direct to implant breast reconstruction requires less manipulation compared to other surgical methods. These factors may explain the lower risk of NAC necrosis. Therefore, the use of endoscopy or robotics, enabling minimal access surgery, may facilitate smaller and strategically placed incisions away from the NAC, effectively reducing the rate of NAC necrosis.

A systematic review^33^ found that IMF incisions were not superior to other types of incisions in NSM. However, because the approach is similar to that of M-NSM, which does not cut the breast envelope, a subgroup analysis was performed on patients who underwent C-NSM with an IMF incision. No significant differences were seen in the incidence of skin flap necrosis, NAC necrosis, bleeding, hematoma, seroma, or implant loss between the 2 groups. Previous research on the association between NSM complications and incision type revealed that IMF incisions resulted in significantly lower rates of NAC necrosis than other approaches.^5,30,34,35,36^ Furthermore, shorter lengths of IMF incisions have been associated with a lower risk of ischemic complications.^37^ Compared to the C-NSM group, which used IMF incisions, the M-NSM group had a significantly higher incidence of breast infections. However, these infections were mostly minor and did not lead to serious complications, as there was no significant difference in implant loss between the 2 groups.

Endoscopy-assisted or robot-assisted NSM has the advantage of reducing the length of skin incision compared to C-NSM. Additionally, an instrument-based approach enables the hiding of skin incisions in the IMF or periareolar area. Shorter and more concealed incisions may improve aesthetic and psychological outcomes and potentially reduce pain levels experienced by patients. However, due to the retrospective nature of the study, pain severity was not evaluated. Nonetheless, Moon et al^37^ observed a reduction in immediate postoperative pain with robotic assistance, indicating that shorter incision lengths could contribute to this effect. Furthermore, the shorter incision line can lead to a shorter suturing time, which may shorten the duration of surgery and reduce the surgeon’s workload.^38^ The multicenter, retrospective design of this study and the variety of skin incision types used resulted in small sample sizes for each category, limiting the statistical power to draw firm conclusions about this variable. This limitation hinders more detailed analyses but reflects real-world data, allowing for a broader comparison of complication rates across different incisions with those of M-NSM.

In the M-NSM group, the incidence of infection was higher than that in C-NSM group, whereas the incidence of seroma was significantly lower. This can be attributed to the frequent use of implants, acellular dermal matrixes, and advanced energy devices. The relatively more frequent use of implants and acellular dermal matrixes in the M-NSM group is expected to contribute to a higher infection rate.^39,40,41,42^ Energy devices facilitate efficient tissue resection and hemostasis during surgery, which can help reduce the frequency of seroma.^43,44^ Therefore, in this study, the observed differences in infection and seroma rates between groups were likely due to the variations in surgical instruments used during the procedures.

### Limitations

This study has several limitations that should be considered. First, the retrospective design may introduce selection bias. Nonetheless, the uniform application of selection criteria for NSM procedures across both C-NSM and M-NSM groups, which exclude patients with nipple or skin involvement, likely minimizes the impact of this bias on the results. Second, the retrospective nature of the study often results in missing critical data, as analyses are conducted postintervention. Efforts to collect data on patients’ bra cup sizes before surgery were hampered, with approximately 80% of these data missing and subsequently excluded. Additionally, data on breast ptosis were absent for about 36% of cases. It was also challenging to determine whether seromas originated in the breast or the axilla. Despite these limitations regarding data sufficiency, the study still managed to achieve significant findings. Another constraint was the nonrandom assignment of participants to the 2 study groups. However, the study involved more than 1500 patients from more than 20 centers in South Korea, and statistical adjustments were made for major factors that could influence the results. The findings of this study could serve as a measure to anticipate the results of ongoing clinical trials (eg, the Prospective Study of Mastectomy With Reconstruction Including Robot Endoscopic Surgery [MARRES] study and the Robot-assisted vs Open NSM With Immediate Breast Reconstruction [ROM] study).^45^ Third, this retrospective study, focusing on early data on endoscopic and robotic surgery, had a relatively small number of M-NSM cases compared to C-NSM, although the number aligns with those reported in other studies. The KoREa-BSG is collecting more data through the prospective MARRES study.^45^ Sentinel lymph node biopsy was also more common in M-NSM, reflecting the surgical consensus at the time that robotic NSM should be limited to clinically node-negative breast cancer.^46^ However, clinical indications for robotic surgery in breast cancer have expanded recently, including axillary lymph node dissection.^47,48^ Additionally, this retrospective study involved surgeons from 21 institutions and primarily early robotic surgery, possibly introducing bias and skewing results toward worse outcomes. However, no significant differences were found compared to studies from just 4 institutions, suggesting that these findings might offer a broader perspective by incorporating diverse early-stage experiences.^32^ Recent advancements in robotic systems have expanded the surgical options for M-NSM, including various flap surgeries, thus overcoming previous limitations in selection criteria.^49,50,51,52^

## Conclusions

In conclusion, our study found no significant differences in the incidence and severity of postoperative complications between C-NSM and M-NSM. M-NSM, which uses endoscopic or robotic-assisted techniques, provides benefits such as less visible scarring, smaller incisions, and a reduced risk of NAC necrosis. The similar complication rates suggest that both C-NSM and M-NSM may be equally safe options. Therefore, the choice of surgical approach should be tailored to patient preferences and individual needs.

## Notes

Supplement 1. eFigure 1. Scoring system (A-F) for the degree of nipple or areolar necrosis after nipple-sparing mastectomy. eFigure 2. Scoring system for degree of skin necrosis after nipple-sparing mastectomy based on the involved area and depth of skin necrosis. eTable. Comparison of patients who underwent conventional nipple-sparing mastectomy (C-NSM) using inframammary fold (IMF) incision or minimal-access NSM (M-NSM)

Supplement 2. The Korea Robot-endoscopy Minimal Access Breast Surgery Study Group members

Supplement 3. Data sharing statement
