# Supplementary material for: Minimal Access vs Conventional Nipple-Sparing Mastectomy
Source: JAMA Surg. 2024 Aug 14;159(10):1177–86. doi: 10.1001/jamasurg.2024.2977 (PMC11325243; doi:10.1001/jamasurg.2024.2977)
Supplement: Supplement 1. — eFigure 1. Scoring system (A-F) for the degree of nipple or areolar necrosis after nipple-sparing mastectomy. eFigure 2. Scoring system for degree of skin necrosis after nipple-sparing mastectomy based on the involved area and depth of skin necrosis. eTable. Comparison of patients who underwent conventional nipple-sparing mastectomy (C-NSM) using inframammary fold (IMF) incision or minimal-access NSM (M-NSM) [file jamasurg-e242977-s001.pdf]

Supplemental Online Content

Kim JH, Ryu JM, Bae SJ, et al; the Korea Robot-endoscopy Minimal Access Breast Surgery Study Group. Minimal access vs conventional nipple-sparing mastectomy. *JAMA Surg*. Published online August 14, 2024. doi:10.1001/jamasurg.2024.2977

eFigure 1. Scoring system (A-F) for the degree of nipple or areolar necrosis after nipple-sparing mastectomy.

eFigure 2. Scoring system for degree of skin necrosis after nipple-sparing mastectomy based on the involved area and depth of skin necrosis.

eTable. Comparison of patients who underwent conventional nipple-sparing mastectomy (C-NSM) using inframammary fold (IMF) incision or minimal-access NSM (M-NSM)

This supplemental material has been provided by the authors to give readers additional information about their work.

© 2024 Kim JH et al. *JAMA Surgery.*

eFigure 1. Scoring system (A-F) for the degree of nipple or areolar necrosis after nipple-sparing mastectomy.

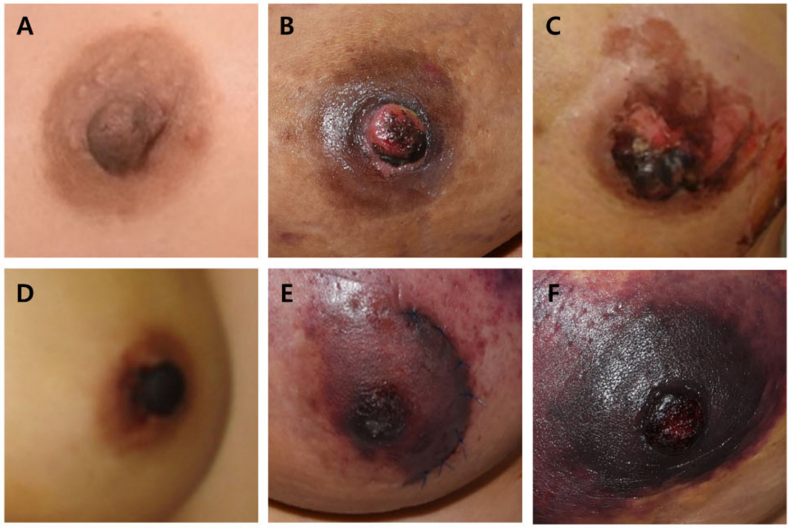

Score	Degree of nipple or areolar ischemia/necrosis
A	No ischemia
B	Partial nipple or areolar necrosis
C	Partial nipple and areolar necrosis
D	Only nipple necrosis
E	Total nipple and partial areolar necrosis
F	Total nipple and total areolar necrosis

eFigure 2. Scoring system for degree of skin necrosis after nipple-sparing mastectomy based on the involved area and depth of skin necrosis.

© 2024 Kim JH et al. *JAMA Surgery.*

Score	Area (%)	Degree	Depth of skin necrosis
0	<1	A	No evidence of skin necrosis
1	1-10	B	Only bruising due to impaired perfusion or ischemic injury
2	11-30	C	Partial thickness of skin necrosis
3	>30	D	Full thickness of skin necrosis

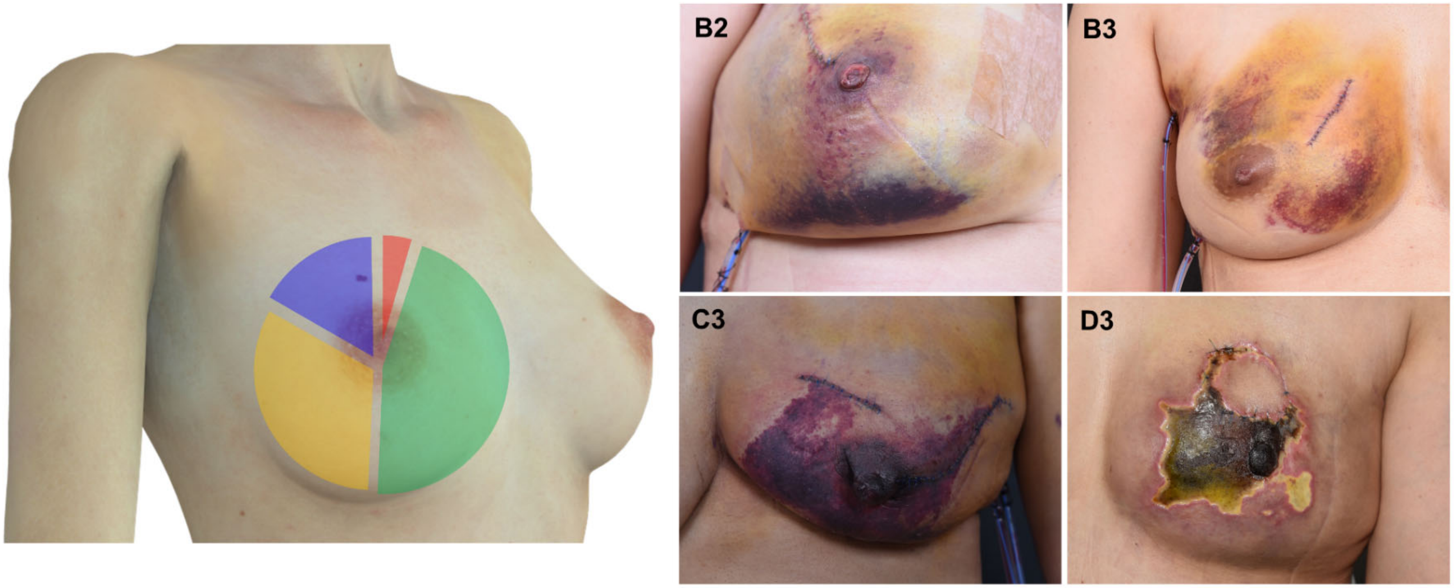

eTable 1. Comparison of patients who underwent conventional nipple-sparing mastectomy (C-NSM) using inframammary fold (IMF) incision or minimal-access NSM (M-NSM)

	C-NSM using IMF incision (N=332)	M-NSM (N=227)	*P*-value

© 2024 Kim JH et al. *JAMA Surgery.*

Postoperative complications		Mean or Frequency	SD[*] or %	Mean or Frequency	SD or %	
Breast skin flap necrosis	A	315	94.88	211	92.95	.0821
	B	3	0.90	4	1.76	
	C	7	2.11	10	4.41	
	D	5	1.51	0	0.00	
	Unknown	2	0.60	2	0.88	
Nipple-areolar complex necrosis	No ischemia	317	95.48	200	88.11	.6300
	Ischemia or necrosis	7	2.11	5	2.20	
	Unknown	8	2.41	22	9.69	
Breast infection	No	329	99.1	208	91.63	<.0001
	Minor infection	0	0.00	13	5.73	
	Severe infection	2	0.60	5	2.20	
	Unknown	1	0.30	1	0.44	
Postoperative bleeding	No	321	96.69	222	97.80	.4631
	Yes	9	2.71	4	1.76	
	Unknown	2	0.60	1	0.44	
Hematoma	No	320	96.39	220	96.92	.7338
	Yes	12	3.61	7	3.08	
	Unknown	0	0.00	0	0.00	
Postoperative seroma	No	287	86.45	205	90.31	.1487
	Yes	44	13.25	21	9.25	
	Unknown	1	0.30	1	0.44	
Duration of seroma formation (days)		18.79	17.41	13.93	26.61	.5315
Total volume of aspirated seroma (mL)		86.73	107.62	60.80	53.00	.2373
Implant loss	No	322	96.99	221	97.36	.7076

© 2024 Kim JH et al. *JAMA Surgery.*

	Yes	9	2.71	5	2.20	
	Unknown	1	0.30	1	0.44	
Initial incision size (mm)		52.52	6.62	45.33	11.49	<.0001
Final incision size (mm)		83.62	13.16	48.61	11.89	<.0001

*SD, standard deviation

© 2024 Kim JH et al. *JAMA Surgery.*
