# Supplementary material for: Minimal Access vs Conventional Nipple-Sparing Mastectomy
Source: JAMA Surg. 2024 Aug 14;159(10):1177–86. doi: 10.1001/jamasurg.2024.2977 (PMC11325243; doi:10.1001/jamasurg.2024.2977)
Supplement: Supplement 2. — The Korea Robot-endoscopy Minimal Access Breast Surgery Study Group members [file jamasurg-e242977-s002.pdf]

Supplemental Online Content: Nonauthor Collaborators

*First name, last name, and suffix (if applicable) are required and will appear in PubMed.

***Group Name(s): Korea Robot-endoscopy Minimal Access Breast Surgery Study Group (KoREa-BSG)**

***First Name and Middle**	***Last Name**	***Suf fix (eg, Jr,**	Academic Degrees	Institution	Location (city, state/province, country)	Role or Contribution, eg, chair, principal	Group (if more than 1 Group listed in the byline) and/or Subgroup (eg, Steering Committee)
Jeea	Lee		MD	Department of Surgery, Uijeongbu Eulji Medical Center, Eulji University	Uijeongbu, Republic of Korea	principal investigator	Korea Robot-endoscopy Minimal Access Breast Surgery Study Group (KoREa-BSG)
Sae Byul	Lee		MD, PhD	Department of Surgery, University of Ulsan College of Medicine, Asan Medical Center	Seoul, Republic of Korea	principal investigator	Korea Robot-endoscopy Minimal Access Breast Surgery Study Group (KoREa-BSG)
Young Woo	Chang		MD, PhD	Division of Breast and Endocrine Surgery, Department of Surgery, Korea University [illegible]	Ansan, Republic of Korea	principal investigator	Korea Robot-endoscopy Minimal Access Breast Surgery Study Group (KoREa-BSG)
Jee Ye	Kim		MD, PhD	Division of Breast Surgery, Department of Surgery, Yonsei University College of [illegible]	Seoul, Republic of Korea	principal investigator	Korea Robot-endoscopy Minimal Access Breast Surgery Study Group (KoREa-BSG)
