# Supplementary material for: Minimal Access vs Conventional Nipple-Sparing Mastectomy
Source: JAMA Surg. 2024 Aug 14;159(10):1177–86. doi: 10.1001/jamasurg.2024.2977 (PMC11325243; doi:10.1001/jamasurg.2024.2977)
Supplement: Supplement 3. — Data sharing statement [file jamasurg-e242977-s003.pdf]

Data Sharing Statement

Kim. Minimal Access vs Conventional Nipple-Sparing Mastectomy. *JAMA Surg*. Published August 14, 2024. doi:10.1001/jamasurg.2024.2977

Data
Data available: Yes
Data types: Deidentified participant data
How to access data: The datasets are available from the corresponding author (j.lee@knu.ac.kr) upon reasonable request.
When available: With publication

Supporting Documents
Document types: None

Additional Information
Who can access the data: The datasets generated and/or analyzed during the current study are not publicly available. However, they are available from the corresponding author (j.lee@knu.ac.kr) upon reasonable request.
Types of analyses: Only research purpose
Mechanisms of data availability: after approval of a proposal
